# Influence of Thiazolidine-2,4-Dione Derivatives with Azolidine or Thiosemicarbazone Moieties on *Haemophilus* spp. Planktonic or Biofilm-Forming Cells

**DOI:** 10.3390/molecules24061051

**Published:** 2019-03-17

**Authors:** Nazar Trotsko, Urszula Kosikowska, Sylwia Andrzejczuk, Agata Paneth, Monika Wujec

**Affiliations:** 1 Department of Organic Chemistry, Faculty of Pharmacy with Medical Analytics Division, Medical University of Lublin, 4A Chodźki, 20-093 Lublin, Poland; agata.paneth@umlub.pl (A.P.); monika.wujec@umlub.pl (M.W.); 2 Department of Pharmaceutical Microbiology with Laboratory for Microbiological Diagnostics, Faculty of Pharmacy with Medical Analytics Division, Medical University of Lublin, 1 Chodźki, 20-093 Lublin, Poland; urszula.kosikowska@umlub.pl (U.K.); sylwia.andrzejczuk@umlub.pl (S.A.)

**Keywords:** *Haemophilus* spp., thiazolidine-2,4-dione based azolidine and chlorophenylthiosemicarbazone hybrids, anti-planktonic and antibiofilm activity, anti-adhesive properties

## Abstract

Biofilm, naturally formed by microorganisms as integrated surface-bound communities, is one of the reasons for the development of antimicrobial resistance. *Haemophilus* spp. are common and representative opportunistic Gram-negative rods forming from the upper respiratory tract microbiota. The aim of this paper was to evaluate the influence of thiazolidine-2,4-dionebased azolidine and chlorophenylthiosemicarbazone hybrids against both planktonic and biofilm-forming *Haemophilus* spp. cells. The in vitro activity against planktonic and biofilm-forming cells of the tested compounds were evaluated by using the broth microdilution method. These activities were detected against reference and clinical strains of *Haemophilus* spp. on the basis of MICs (minimal inhibitory concentrations) and MBICs (minimal biofilm inhibitory concentrations). In addition, anti-adhesive properties of these compounds were examined. The target compounds showed potential activity against planktonic cells with MIC = 62.5–500 mg/L and biofilm-forming cells with MBIC = 62.5–1000 mg/L. The observed anti-adhesive properties of the tested compounds were reversible during long-term incubation in a lower concentration of compounds.

## 1. Introduction

One of the main problems of the effectiveness of antimicrobial therapy is the development of resistance to agents among both bacteria and fungi. It is an adaptive mechanism for self-survival.

Most bacteria and fungi naturally form biofilms as integrated surface-bound communities, which are important to survive in the host’s body and in the natural environment. It is one of the reasons for the development of antimicrobial resistance. These structures are dynamic, integrated, both genetically and physiologically heterogenous, sedimentary multicellular communities, embedded in an extracellular biopolymer matrix [1]. Biofilms account for more than 80% of infectious diseases, which cause unpleasant wounds, ulcers, and lesions of the skin, mucous, membranes, and internal organs [2,3]. Additionally, biofilms cause chronic infections in tissues or by developing on the surfaces of medical biomaterials.

*Haemophilus* spp. are common and representative opportunistic Gram-negative rods, representing bacteria forming from the upper respiratory tract microbiota. These fastidious bacteria, which grow in microaerophilic conditions (higher level of CO_2_), play a role in preventing the establishment of potential pathogens and are important for the human body condition [4,5]. Many species of haemophili normally reside in the airways and they can, rarely, cause infection in the respiratory tract, which can spread to other organs.

*Haemophilus influenzae* and some of the other *Haemophilus* species are commonly encountered in clinical microbiology laboratories and demonstrate a wide range of pathogenicity, from life-threatening invasive disease to respiratory infections to a nonpathogenic, commensal lifestyle. It is the most pathogenic bacteria colonizing the mucous membranes of the respiratory tract of young children or, sporadically, of elderly people. *H. influenzae* is frequently associated with different diseases (e.g., otitis media in children, sinusitis, conjunctivitis, chronic bronchitis, and pneumonia) and cause exacerbations in adults with chronic obstructive pulmonary disease [6]. Some of them, like bloodstream infections, are very serious.

In contrast to the accepted pathogenicity of *H. influenzae*, *H. parainfluenzae* is an opportunistic bacteria, with low pathogenicity that is highly adapted to the human airways [7,8]. Both *H. influenzae* and *H. parainfluenzae* occasionally, especially in immunocompromised people or after translocation in the body, can cause opportunistic acute, chronic, invasive, or non-invasive infections [9,10].

Like many opportunistic pathogens inhabiting mammalian mucosal surfaces, non-typeable *H. influenzae* (NTHi) forms multicellular biofilm communities, both in vitro and in various infection models. These characteristics were also shown among *H. parainfluenzae* species isolated from healthy people, and from patients with various diseases [11,12]. It is known nowadays that these microorganisms may form a biofilm, which is the most prevalent mode of bacterial growth in nature and is a virulence determinant, which contributes to recurrent or chronic infections. Bacterial adhesion and their ability to growth in biofilm structure is a main problem in many environments (including biomedical, domestic, and natural) and for virulence properties of microorganisms with low pathogenicity. During the initial stage of biofilm formation (known as “early biofilm”), the adhesive properties of bacterial cells cause attachment to a colonizable surface (e.g., human tissue). Next, bacterial cells cause irreversible attachment to a surface, and a biofilm is established and matures (“mature biofilm”) [13].

Both *H. influenzae* and *H. parainfluenzae*, as well as other haemophili diseases, are treated with various antimicrobials, mainly with beta-lactam antibiotics (including cephalosporins like ceftriaxone, cefotaxime, or cefuroxime, as well as amoxicillin-clavulanate), macrolides (e.g., azithromycin and clarithromycin), and fluoroquinolones. Which ones can be used depends on the location and severity of the infection and of the results of susceptibility tests. The CDC (Centers for Disease Control and Prevention) has guidelines for chemoprophylaxis for close contacts of *H. influenzae* type b (Hib) cases, but does not have recommendations for the treatment of infections caused by species other than *H. influenzae* species [14]. The problem is observed for infections caused by other species of the genus *Haemophilus*, including opportunistic pathogens like NTHi and *H. parainfluenzae*, which, usually, can be the result of the local spread of microorganisms from the nasopharynx or after their translocation in the body. In the case of opportunistic infections caused by these bacteria, the cells’ properties and the ability to grow a biofilm structure are important pathogenicity factors. Due to the increasing drug resistance of these bacteria, especially to beta-lactam antibiotics (the production of beta-lactamases due to changes in the cell wall structure) and the possibility of biofilm formation, it is necessary to look for new agents effective against these bacteria, both against planktonic and biofilm-forming cells [15,16]. Thiazolidine-2,4-dione (TZD) is a heterocyclic ring system, with multiple applications in medicinal chemistry through their wide range of biological activity. TZDs possess antidiabetic (glitazones drugs), aldose reductase inhibitory, anticancer, antibacterial, antifungal, and anti-inflammatory activities [17,18].

In a previous paper we described the antibacterial activity of TZD derivatives with azolidine and chlorophenylthiosemicarbazone moieties against aerobically-growing bacteria, like staphylococci or other Gram-positive microorganisms [19,20]. Evaluation of the influence of these compounds on the biofilm-forming and planktonic cells of fastidious and microaerophilic bacteria may be additional information required for the complete evaluation of their antibacterial potential. Especially as there are a few papers about anti-biofilm activity for TZD derivatives against both bacteria and fungi [21,22,23,24,25,26,27,28]. All of these derivatives are a group of 5-alkylidenethiazolidine-2,4-diones with C_6_–C_12_ alkyl chains. Most of the active compounds among this group, namely 5-octylidene-1,3-thiazolidine-2,4-dione (Thiazolidindione-8), inhibit fungal (*Candida albicans*) biofilm [22] and *Cryptococcal* biofilm [27] formation, and showed activity against *Propionibacterium acnes* biofilm [28].

The aim of this paper was to evaluate the influence of TZD-based azolidine and chlorophenyl thiosemicarbazone hybrids against both planktonic and biofilm-forming *Haemophilus* spp. cells. According to our knowledge, it is the first study about the effect of tested compounds against these fastidious bacteria with very specific growth requirements.

## 2. Results and Discussion

### 2.1. Chemistry

The objects of the present research were two series of previously obtained TZD-based hybrids [19,20]. The first group of compounds was a series A (Table 1) that consisted of TZD-based azolidine hybrids. Substances of series A were characterized by two nitrogenous five-membered heterocyclic rings. Compounds of series A were obtained by three-step synthesis using (2,4-dioxothiazolidin-5-yl/ylidne)acetic acids as starting materials. Target TZD-based azolidine hybrids were synthesized by the reaction of (2,4-dioxothiazolidin-5-yl/ylidene)acetic acid chlorides with 5-(hydroxybenzylidene)azolidine derivatives. The second group of research compounds was a series B (Table 2) that consisted of TZD-based chlorophenylthiosemicarbazone hybrids. Compounds of series B were obtained also by a three-step procedure, starting with appropriate (2,4-dioxothiazolidin-5-yl/ylidene)acetic acids and connection with the chlorophenylthiosemicarbazide fragment by the reaction of condensation in the last step of the synthesis.

### 2.2. Activity Assay against Planktonic and Biofilm-Forming Cells

The in vitro activity of the tested compounds against planktonic and biofilm-forming cells of reference and clinical isolates of *Haemophilus* spp. were evaluated by using the broth microdilution method. Among the 62 evaluated compounds, of TZD-based hybrids consisting of series A and B, 39 compounds were inactive against both planktonic and biofilm-forming cells of *Haemophilus* spp. in a concentration more than 1000 mg/L. Three compounds had a very low inhibitory effect (MIC = 500–1000 mg/L) on the growth of planktonic and biofilm-forming cells of the tested bacteria. The other twenty compounds (twelve of series A and eight of series B) had a moderate effect on the growth, with MICs ranging from 31.25 to 250 mg/L, and the biofilm formation, with MBICs ranging from 62.5 to 250 mg/L.

Compounds **34A**, **41A**, **56A**, and **60A** showed activity at MIC value of 125 mg/L against reference strains and planktonic cells of *Haemophilus* spp. The activity of these compounds against biofilm-forming cells was at MBIC = 250 mg/L. Substances **54A** and **57A** showed activity with MIC = 62.5–125 mg/L against planktonic cells of reference strains. The activity of the **54A** compound against biofilm-forming cells was with MBIC value of 250 mg/L and for **57A** with MBIC value of 1000 mg/L. The activity of compounds of series A against biofilm-forming cells was highest for the clinical isolate of *H. parainfluenzae* 201. The compounds **39A**, **52A**, **54A**, **56A**, **57A**, **59A**, and **64A** showed this activity at MBIC = 125 mg/L. This value was two-fold lower than for reference compound gentamycin. Compound **47A** showed activity against *H. parainfluenzae* 201 biofilm-formation at 62.5 mg/L, and it had very low activity against other *Haemophilus* spp. Worth noticing is that compound **60A**, and likewise **47A**, showed activity against *H. parainfluenzae* 201 growth in biofilm at 62.5 mg/L. Moreover, compounds **47A** and **60A** showed four-fold better activity against *H. parainfluenzae* 201 biofilm formation than gentamycin, and comparable activity with reference compound cefuroxime. Compound **60A** had the best activity against both planktonic and biofilm-forming cells of *Haemophilus* spp. amongst evaluated compounds of series A.

All compounds of series A with moderate inhibitory antibacterial effect had, in its structure, an electron withdrawing group (Br or Cl) (compounds **34A**, **41A**, **47A**, **54A** and **60A**) in the 2 position of the phenyl ring or were without substituent in this position (compounds **57A** and its *meta* isomer **56A**). Introducing an electron donating group (methoxy and ethoxy group) into the structure significantly decreased activity (compounds **39A**, **51A**, **52A**, **58A** and **59A**). The type of azolidine heterocycle attached to the benzylidene fragment did not have a major impact on the activity, as well as the presence of the double bond in position 5 of the TZD system.

Compounds of series B, generally, showed very low activity against planktonic and biofilm-forming cells, for the three reference species and the three clinical isolates of haemophili. It is worth paying attention to the clinical *H. parainfluenzae* 201 isolate. Against this strain, the most active were compounds of series B (**19B**, **21B**, **23B**, **24B**, **26B**, and **29B**)—with MIC value of 100–125 mg/L. The **22B** compound was active against planktonic cells of the *H. parainfluenzae* 201 isolate, with MIC = 31.25 mg/L.

To determine the power of the tested compounds (series A and series B) as agents with anti-biofilm activity, they were compared with the activity against planktonic cells on the basis of the MBIC/MIC ratio. The results, included in Table 3, showed that the MBIC/MIC ratio ranged 1–8 in the case of *H. parainfluenzae* ATCC 33392, 1–16 in the case of *H. parainfluenzae* ATCC 51505, and 1–4 in the case of *H. influenzae* ATCC 10211. In the case of clinical isolate *H. parainfluenzae* 201, the MBIC/MIC ratio ranged within 0.0625–0.5 for compounds of series A, and within 0.5–32 for derivatives of series B. As shown by the MBIC/MIC ratio equal <1 in some cases, the activity against biofilm-forming cells was higher compared to the activity against planktonic cells of the same species.

**Figure 1:**
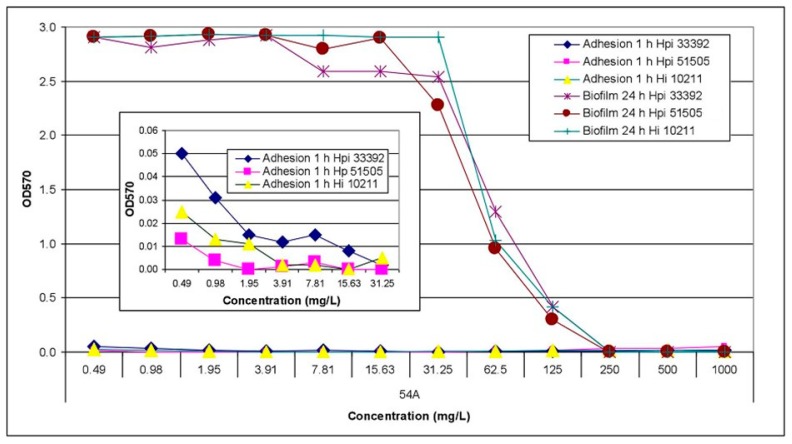
Anti-adhesive and antibiofilm activity of compound **54A** against reference haemophili species, assessed on the basis of OD_570_ values. Hpi—*Haemophilus parainfluenzae*, Hi—*Haemophilus influenzae*.

The most anti-adhesive properties of tested compounds **54A**, **56A**, **59A**, and **60A** with potential antibiofilm activity (MBIC = 62.5–250 mg/L) against reference species of *H. parainfluenzae* and *H. influenzae* were detected. As was shown on the **54A** compound example (Figure 1), the inhibitory effect against the first step of biofilm formation of tested compounds was dependent on both the compound and their concentration. The anti-adhesive properties were reversible during long-term incubation of bacteria in the presence of lower concentrations of compounds. After 24 h of incubation, biofilm was formed independently to the initial inhibition of the adhesion of bacteria to the polystyrene surface.

Among the compounds (**54A**, **56A**, **59A**, and **60A**) with the most anti-adhesive properties, all substances were derivatives of (2,4-dioxothiazolidin-5-ylidene)acetic acid with a double bond in the 5 position of the TZD ring, and mainly contained rhodanine ring (compounds **56A**, **59A** and **60A**) in its structure. The substituent in the 2 position of the phenyl ring did not play a significant role in the anti-adhesive properties; additionally, the isomerization in the phenyl ring (*meta* isomer compound **56A**) seemed to be negligible.

It is known that adhesion is the key stage of biofilm formation and microbial colonization, and it is an essential element of microbial virulence. The ability of microorganisms to adhere to eukaryotic cells is a pathogenic factor that is considered to be the first stage of infection. It depends, to a large extent, on the characteristics of prokaryotic cells, such as the production of extracellular factors (e.g., mucus) and the specific features of the surfaces (e.g., their structure, hydrophobicity, and presence of receptors). They can affect the adhesive properties of microorganisms to abiotic and biotic surfaces and the possibility of biofilm formation [29]. Detected MBIC values were often lower compared to the MICs after 24 h of culture (Table 3). Additionally, the adhesion of tested bacteria was inhibited at lower concentrations of the tested compounds. These values indicate that the test substances and both the antibiotics inhibited the biofilm formation by the cells of the tested strains, at concentrations that did not affect their growth. This suggests that in the case of *Haemophilus* spp., the activity of these compounds was related to their effect on the initial phases of biofilm formation, as well as adhesion properties and adherence of cells to the solid surface.Found that cefuroxime, at sub-inhibitory concentrations below 50% of MIC, inhibited the adhesion of various *H. influenzae* strains to cheek epithelial cells [30]. The influence of sub-inhibitory concentrations of various antimicrobial substances on biofilm formation in the early stages of its formation was demonstrated by many authors (e.g., [31]). Incubation of *Escherichia coli* with sub-inhibitory concentrations of ciprofloxacin blocked bacterial adhesion [32]. The use of other antibiotics (e.g., piperacillin with tazobactam) at a concentration of 1/2 MIC caused inhibition of *Pseudomonas aeruginosa* biofilm formation on polystyrene plates [33]. This phenomenon was caused by the reduction of the cell adhesion capacity as a result of morphological changes, such as cell elongation. Additionally, co-trimoxazole caused the greatest inhibition of adhesion at 1/2 MIC of *E. coli* strain, when compared with the controls, followed by ceftazidime [34].

The biofilm structure, formed by bacteria and fungi, is an important problem related to the use of antimicrobials [35]. Several approaches have been studied to prevent the adhesion to various surfaces (natural or synthetic) and of microorganisms growing in biofilm. The ability of newly synthesized compounds to affect microbial adherence and biofilm formation may be an important criterion in selecting the ones, for the design of the substances, with the improved antimicrobial activity. It is known that low (sub-MICs) concentrations of antimicrobials do not kill bacteria, but they are capable of changing their biochemical and structural properties. The final effect is a reduction of bacterial pathogenicity by compounds’ interference with the important aspects of bacterial properties, including adherence, fimbriation, or motility.

## 3. Materials and Methods

### 3.1. Chemistry

In this work, the following TZD-based azolidine and chlorophenylthiosemicarbazone hydrids were used [19,20]. TZD-based azolidine hybrids seria consisted of 38 compounds (series A). But TZD-based chlorophenylthiosemicarbazone hybrids seria consisted of 24 compounds (series B). The physicochemical and spectral characteristic of the compounds of series A and B, as well as the rationale for the chemical combination of the above mentioned structures in one compound, was described in previously published papers [19,20].

### 3.2. Bacterial Strains

The haemophili reference species from American Type Culture Collection (ATCC)—*H. influenzae* ATCC 10211, *H. parainfluenzae* ATCC 33392, and *H. parainfluenzae* ATCC 51505 were included. Besides, four clinical isolates of *H. parainfluenzae* from the collection of the Department of Pharmaceutical Microbiology with Laboratory for Microbiological Diagnostics of Medical University of Lublin were used. Bacterial strains were suspended in fresh TSB + HTMS medium and standardized with OD_600_ equivalent to the 0.5 McFarland standard (OD_600_ = 0.08 ± 0.02), using the microplate reader ELx800 (BioTek, Instruments, Winooski, VT, USA). For each measurement, the bacterial suspensions were prepared independently.

### 3.3. Determination of the Minimal Inhibitory Concentration (MIC) of Tested TZD-Based Azolidine and Chlorophenyl Thiosemicarbazone Hybrids

Antibacterial activity of TZD-based azolidine hybrids and TZD-based chlorophenylthiosemicarbazone hybrids, as well as of reference agents against planktonic cells of bacteria, was screened with *Haemophilus* spp. reference strains and clinical isolates by the broth microdilution method, using 96-well polystyrene microplates (96F-Well Microplates, Thermo Scientific™ Nunc™ Brand Product, Roskilde, Denmark). The medium was prepared from TSB (trypticase soy broth; Biocorp, Warsaw, Poland) and 0.4% HTMS (*Haemophilus* test medium supplemented with growth factors for haemophili—25 mg/L of NAD and 15 mg/L of hematin, Oxoid, UK) and marked as TSB + HTMS. Stock solutions of TZD-based hybrids at a concentration of 50 mg/mL in dimethyl sulfoxide (DMSO, Sigma Aldrich, St. Louis, MO, USA) were prepared and diluted in TSB + HTMS medium, in the range of concentration depending on the experiment. The activity of the compounds tested against planktonic cells was determined as the MIC, defined as the lowest concentration of the antimicrobial agents that inhibits visible growth of bacteria, according to the EUCAST procedure [36] with some modifications [37]. The medium with bacteria and without the tested compounds added served as a growth control. The wells with a two-fold dilution of the tested compounds added to TSB + HTMS broth and without bacterial suspension served as compound control (negative control) and with antimicrobials (gentamicin and cefuroxime) as reference agents (positive control). The wells with only TSB + HTMS broth and without bacterial suspension was a medium sterility control. All controls were incubated under the same conditions in three replications for a blank value. Each measurement was performed in triplicate, and both positive and negative controls were kept. During the experiments, 198 μL of TSB + HTMS medium, with and without a series of two-fold dilutions of the tested compounds or reference compounds, were inoculated with 2 μL of the standardized microbial suspension (total volume per each well—200 μL/well), and then incubated for 24 h at 35 °C in the presence of about 5% CO_2_. Absorbance was measured as an OD_570_ using a microplate reader. Each measurement was made in triplicate.

### 3.4. Determination of the Minimal Biofilm Inhibitory Concentration (MBIC) of Tested TZD-Based Azolidine and Chlorophenyl Thiosemicarbazone Hybrids

In order to assay the effect on *Haemophilus* spp. of biofilm formation, the method based on staining with 0.1% crystal violet, as described earlier [38], was used. The activity of the tested compound against biofilm-forming cells was determined on the basis of MBIC (minimal biofilm inhibitory concentration), defined as the lowest concentration of the tested compound at which the biofilm formation was inhibited [39], and in the concentration at which bacterial growth was observed [40]. After overnight incubation of bacterial isolates under microaerophilic atmosphere and conditions described above, the medium above the culture was decanted and then the plates were washed extensively several times with distilled water to remove nonadherent or loosely adherent cells, dried in inverted position, and stained with 200 µL of 0.1% crystal violet. The plates were left for 15 min at room temperature to stain the cells, then washed extensively under distilled water to remove unbound dye. Next, in order to elicit a response to each of the wells, 200 μL of ethanol alcohol was added and the plates were left at room temperature for 15 min to solubilize the dye into the alcohol. The OD_570_ of the alcohol-–dye solution in each well was read by using a microplate reader (BioTek ELx800). The blank control wells, without or with two-fold dilution of the tested compounds and reference agents added to the same broth but without bacterial suspension, were incubated under the same conditions. OD_570_ values read in these wells were the ODc values, being the reference point for determining the MBIC value.

### 3.5. Determination of the Anti-Adhesive Properties of Tested TZD-Based Azolidine and Chlorophenylthiosemicarbazone Hybrids

The anti-adhesive properties of tested compounds were assessed after 1 h incubation in the conditions and by a method such as the biofilm was done, but after the incubation time of 1 h. Besides the much shorter time of incubation, the assay was carried out according to the biofilm detection procedure (as stated above). After 1 h of incubation, the cultures from microplates were removed, the wells were rinsed and, after drying, stained with 0.1% crystal violet for 10 min. After washing off the excess dye, microwells were poured for 15 min with ethanol. It was very important to determine the OD_570_ values for the same substances without cells. Absorbance values were obtained using a 570 nm wavelength spectrometer (OD_570_).

## 4. Conclusions

The influence of TZD-based azolidine (series A) and chlorophenylthiosemicarbazone (series B) hybrids against both planktonic and biofilm-forming *Haemophilus* spp. cells was evaluated. In addition, anti-adhesive properties of these compounds were examined. Among the tested compounds, twenty derivatives possessed moderate effect on the growth, with MICs ranging from 31.25 to 250 mg/L, and the biofilm formation, with MBICs ranging from 62.5 to 250 mg/L. The compounds **54A**, **56A**, **59A**, and **60A,** with potential antibiofilm activity against reference species of *H. parainfluenzae* and *H. influenzae*, exhibited good anti-adhesive properties. The observed anti-adhesive properties of the tested compounds were reversible during long-term incubation in lower concentration of compounds.

## Figures and Tables

**Table 1:**
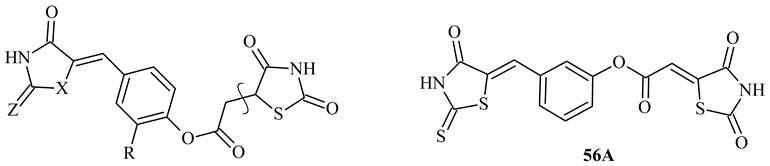
Thiazolidine-2,4-dionebased azolidine hybrids (series A) symbols and structure.

Compound ^1^	Z	X	R	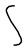	Compound Name
**34A**	O	S	Br	single bond	2-Bromo-4-[(2,4-dioxo-1,3-thiazolidin-5-ylidene)methyl]phenyl (2,4-dioxo-1,3-thiazolidin-5-yl)acetate
**39A**	S	S	OC_2_H_5_	single bond	2-Ethoxy-4-[(4-oxo-2-thioxo-1,3-thiazolidin-5-ylidene)methyl]phenyl (2,4-dioxo-1,3-thiazolidin-5-yl)acetate
**41A**	S	S	Br	single bond	2-Bromo-4-[(4-oxo-2-thioxo-1,3-thiazolidin-5-ylidene)methyl]phenyl (2,4-dioxo-1,3-thiazolidin-5-yl)acetate
**47A**	S	NH	Br	single bond	2-Bromo-4-[(5-oxo-2-thioxoimidazolidin-4-ylidene)methyl]phenyl (2,4-dioxo-1,3-thiazolidin-5-yl)acetate
**51A**	O	S	OCH_3_	double bond	4-[(2,4-Dioxo-1,3-thiazolidin-5-ylidene)methyl]-2-methoxyphenyl (2,4-dioxo-1,3-thiazolidin-5-ylidene)acetate
**52A**	O	S	OC_2_H_5_	double bond	4-[(2,4-Dioxo-1,3-thiazolidin-5-ylidene)methyl]-2-ethoxyphenyl (2,4-dioxo-1,3-thiazolidin-5-ylidene)acetate
**54A**	O	S	Br	double bond	2-Bromo-4-[(2,4-dioxo-1,3-thiazolidin-5-ylidene)methyl]phenyl (2,4-dioxo-1,3-thiazolidin-5-ylidene)acetate
**56A**	-	-	-	-	3-[(4-Oxo-2-thioxo-1,3-thiazolidin-5-ylidene)methyl]phenyl (2,4-dioxo-1,3-thiazolidin-5-ylidene)acetate
**57A**	S	S	H	double bond	4-[(4-Oxo-2-thioxo-1,3-thiazolidin-5-ylidene)methyl]phenyl (2,4-dioxo-1,3-thiazolidin-5-ylidene)acetate
**58A**	S	S	OCH_3_	double bond	2-Methoxy-4-[(4-oxo-2-thioxo-1,3-thiazolidin-5-ylidene)methyl]phenyl (2,4-dioxo-1,3-thiazolidin-5-ylidene)acetate
**59A**	S	S	OC_2_H_5_	double bond	2-Ethoxy-4-[(4-oxo-2-thioxo-1,3-thiazolidin-5-ylidene)methyl]phenyl (2,4-dioxo-1,3-thiazolidin-5-ylidene)acetate
**60A**	S	S	Cl	double bond	2-Chloro-4-[(4-oxo-2-thioxo-1,3-thiazolidin-5-ylidene)methyl]phenyl (2,4-dioxo-1,3-thiazolidin-5-ylidene)acetate
**64A**	S	NH	OC_2_H_5_	double bond	2-Ethoxy-4-[(5-oxo-2-thioxoimidazolidin-4-ylidene)methyl]phenyl (2,4-dioxo-1,3-thiazolidin-5-ylidene)acetate

^1^ Number of compounds were adapted from [19]. A—TZD-based azolidine hybrids.

**Table 2:**
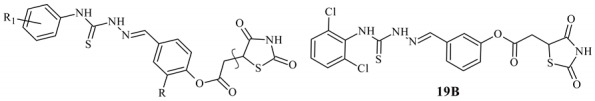
TZD-based chlorophenylthiosemicarbazone hybrids (series B) symbols and structure.

Compound ^1^	R	R_1_	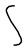	Compound Name
**19B**	-	-	-	3-[{2-[(2,6-Dichlorophenyl)carbamothioyl]hydrazinylidene}methyl]phenyl (2,4-dioxo-1,3-thiazolidin-5-yl)acetate
**20B**	H	2-Cl	single bond	4-[{2-[(2-Chlorophenyl)carbamothioyl]hydrazinylidene}methyl]phenyl (2,4-dioxo-1,3-thiazolidin-5-yl)acetate
**21B**	H	3-Cl	single bond	4-[{2-[(3-Chlorophenyl)carbamothioyl]hydrazinylidene}methyl]phenyl (2,4-dioxo-1,3-thiazolidin-5-yl)acetate
**22B**	H	4-Cl	single bond	4-[{2-[(4-Chlorophenyl)carbamothioyl]hydrazinylidene}methyl]phenyl (2,4-dioxo-1,3-thiazolidin-5-yl)acetate
**23B**	H	2,4-diCl	single bond	4-[{2-[(2,4-Dichlorophenyl)carbamothioyl]hydrazinylidene}methyl]phenyl (2,4-dioxo-1,3-thiazolidin-5-yl)acetate
**24B**	H	2,6-diCl	single bond	4-[{2-[(2,6-Dichlorophenyl)carbamothioyl]hydrazinylidene}methyl]phenyl (2,4-dioxo-1,3-thiazolidin-5-yl)acetate
**26B**	OCH_3_	3-Cl	single bond	4-[{2-[(3-Chlorophenyl)carbamothioyl]hydrazinylidene}methyl]-2-methoxyphenyl (2,4-dioxo-1,3-thiazolidin-5-yl)acetate
**29B**	OCH_3_	2,6-diCl	single bond	4-[{2-[(2,6-Dichlorophenyl)carbamothioyl]hydrazinylidene}methyl]-2-methoxyphenyl (2,4-dioxo-1,3-thiazolidin-5-yl)acetate
**33B**	H	2-Cl	double bond	4-[{2-[(2-Chlorophenyl)carbamothioyl]hydrazinylidene}methyl]phenyl (2,4-dioxo-1,3-thiazolidin-5-ylidene)acetate
**34B**	H	3-Cl	double bond	4-[{2-[(3-Chlorophenyl)carbamothioyl]hydrazinylidene}methyl]phenyl (2,4-dioxo-1,3-thiazolidin-5-ylidene)acetate

^1^ Number of compounds were adapted from [20]. B—TZD-based chlorophenyl thiosemicarbazone hybrids.

**Table 3:** The antibacterial activity of TZD-based azolidine and chlorophenyl thiosemicarbazone hybrids against planktonic and biofilm-forming cells, of reference strains and clinical isolates of *Haemophilus* spp., in vitro under stationary conditions.

	Reference Species									Clinical Isolates											
Compound	*Haemophilus parainfluenzae* ATCC 33392			*Haemophilus parainfluenzae* ATCC 51505			*Haemophilus influenzae* ATCC 10211			*Haemophilus parainfluenzae* 128			*Haemophilus parainfluenzae* 134			*Haemophilus parainfluenzae* 201			*Haemophilus parainfluenzae* 206		
Antibacterial Activity (mg/L)																					
	MIC	MBIC	MBIC/ MIC	MIC	MBIC	MBIC/ MIC	MIC	MBIC	MBIC/ MIC	MIC	MBIC	MBIC/ MIC	MIC	MBIC	MBIC/ MIC	MIC	MBIC	MBIC/ MIC	MIC	MBIC	MBIC/ MIC
**34A**	125	250	2	125	250	2	125	250	2	>1000	>1000	-	>1000	>1000	-	1000	250	0.25	>1000	1000	-
**39A**	500	>1000	-	500	1000	2	500	>1000	-	>1000	>1000	-	>1000	1000	-	250	125	0.5	>1000	1000	-
**41A**	125	250	2	125	250	2	125	250	2	>1000	500	-	>1000	>1000	-	500	250	0.5	>1000	>1000	-
**47A**	500	1000	2	500	1000	2	500	1000	2	>1000	500	-	>1000	1000	-	1000	62.5	0.0625	>1000	1000	-
**51A**	500	500	1	500	500	1	500	500	1	>1000	500	-	>1000	1000	-	1000	>1000	-	>1000	500	-
**52A**	500	1000	2	500	1000	2	500	1000	2	>1000	1000	-	>1000	500	-	250	125	0.5	>1000	500	-
**54A**	125	250	2	125	250	2	62.5	250	4	>1000	>1000	-	>1000	>1000	-	500	125	0.25	>1000	1000	-
**56A**	125	250	2	125	250	2	125	250	2	>1000	>1000	-	>1000	250	-	1000	125	0.125	>1000	250	-
**57A**	125	1000	8	62.5	1000	16	500	>1000	-	>1000	>1000	-	>1000	>1000	-	1000	125	0.125	>1000	250	-
**58A**	250	1000	4	250	1000	4	250	1000	4	>1000	>1000	-	>1000	1000	-	500	>1000	-	>1000	1000	-
**59A**	500	500	1	500	500	1	500	500	1	>1000	>1000	-	>1000	>1000	-	1000	125	0.125	1000	>1000	-
**60A**	125	250	2	125	250	2	125	250	2	>1000	>1000	-	>1000	>1000	-	1000	62.5	0.0625	>1000	1000	-
**64A**	250	>1000	-	1000	>1000	-	1000	>1000	-	>1000	1000	-	>1000	>1000	-	1000	125	0.125	250	>1000	-
**19B**	>1000	500	-	>1000	>1000	-	>1000	500	-	>1000	>1000	-	>1000	500	-	125	125	1	>1000	1000	-
**20B**	>1000	>1000	-	>1000	>1000	-	>1000	>1000	-	>1000	>1000	-	>1000	1000	-	500	1000	2	>1000	1000	-
**21B**	>1000	>1000	-	>1000	>1000	-	>1000	>1000	-	>1000	>1000	-	>1000	1000	-	125	1000	8	>1000	1000	-
**22B**	>1000	>1000	-	>1000	>1000	-	>1000	>1000	-	>1000	>1000	-	>1000	500	-	31.25	1000	32	>1000	1000	-
**23B**	1000	>1000	-	>1000	>1000	-	>1000	>1000	-	1000	1000	1	>1000	1000	-	125	1000	8	>1000	1000	-
**24B**	>1000	>1000	-	>1000	>1000	-	>1000	>1000	-	>1000	>1000	-	>1000	>1000	-	125	1000	8	>1000	1000	-
**26B**	>1000	>1000	-	>1000	>1000	-	>1000	>1000	-	>1000	>1000	-	>1000	1000	-	100	1000	10	>1000	1000	-
**29B**	>1000	>1000	-	>1000	>1000	-	>1000	>1000	-	>1000	>1000	-	>1000	1000	-	100	500	5	>1000	1000	-
**33B**	>1000	>1000	-	>1000	>1000	-	>1000	>1000	-	>1000	>1000	-	>1000	>1000	-	1000	1000	1	>1000	1000	-
**34B**	250	>1000	-	500	>1000	-	500	>1000	-	>1000	>1000	-	>1000	>1000	-	500	250	0.5	>1000	1000	-
**Ge**	250	250	1	1000	250	0.25	500	250	0.5	1000	1000	1	1000	500	0.5	500	250	0.5	1000	500	0.5
**Cef**	125	125	1	500	125	0.25	125	62.5	0.5	500	125	0.25	500	62.5	0.13	250	62.5	0.25	250	125	0.5

MIC—minimal inhibitory concentration, MBIC—minimal biofilm inhibitory concentration, MBIC/MIC—minimal biofilm inhibitory concentration/minimal inhibitory concentration ratio, Ge—gentamycin, Cef—cefuroxime, “-”—not determined.
